# Liquiritigenin Induces Tumor Cell Death through Mitogen-Activated Protein Kinase- (MPAKs-) Mediated Pathway in Hepatocellular Carcinoma Cells

**DOI:** 10.1155/2014/965316

**Published:** 2014-03-11

**Authors:** Di Wang, Jiahui Lu, Yan Liu, Qingfan Meng, Jing Xie, Zhenzuo Wang, Lesheng Teng

**Affiliations:** ^1^College of Life Science, Jilin University, Changchun, Jilin 130012, China; ^2^The State Engineering Laboratory of AIDS Vaccine, Jilin University, Changchun 130012, China

## Abstract

Liquiritigenin (LQ), separated from * Glycyrrhiza radix*, possesses anti-inflammatory, antihyperlipidemic, and antiallergic effects. Our present study aims to investigate the antihepatocellular carcinoma effects of LQ both in cell and animal models. LQ strikingly reduced cell viability, enhanced apoptotic rate, induced lactate dehydrogenase over-release, and increased intracellular reactive oxygen species (ROS) level and caspase 3 activity in both PLC/PRL/5 and HepG2 cells. The expression of cleaved PARP, the hall-marker of apoptosis, was enhanced by LQ. LQ treatment resulted in a reduction of the expressions of B-cell lymphoma 2 (Bcl-2) and B-cell lymphoma-extra large (Bcl-xL), and an increase of the phosphorylation of c-Jun N-terminal kinases (JNK) and P38. LQ-mediated cell viability reduction, mitochondrial dysfunction, apoptosis related protein abnormal expressions, and JNK and P38 activation were partially abolished by N-Acetyl-L-cysteine (a ROS inhibitor) pretreatment. Moreover, LQ suppressed the activation of extracellular signaling-regulated kinase (ERKs) and reduced the translocation of phosphor-ERKs from cytoplasm to nucleus. This antitumor activity was further confirmed in PLC/PRL/5-xenografted mice model. All these data indicate that the antihepatocellular carcinoma effects of LQ are related to its modulation of the activations of mitogen-activated protein kinase (MAPKs). The study provides experimental evidence supporting LQ as a potential therapeutic agent for hepatocellular carcinoma treatment.

## 1. Introduction

Hepatocellular carcinoma (HCC), one of the most common cancers in the whole world, causes nearly 600,000 deaths annually [1]. Patients who suffer from HCC are often diagnosed at a late stage and die within 7-8 months after diagnosis [1]. For years, the standard chemotherapy and radiotherapy for HCC patients remains disappointing [2]. Efforts have been made but no satisfactory drugs were manufactured over the past decade. Novel therapeutic agents for curing HCC are still high in demand.

**Figure 1:**
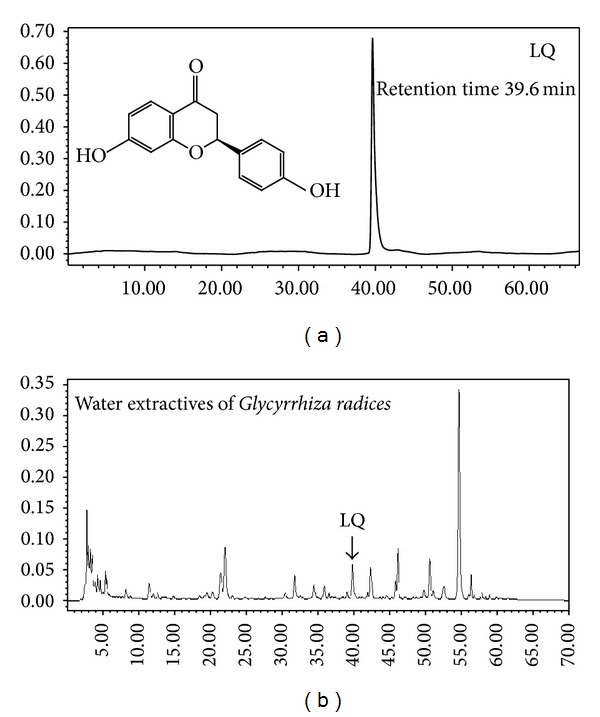
Chemical structure of Liquiritigenin (LQ) (a) and HPLC chromatograms of LQ in water extraction of* Glycyrrhiza radix* (b).

Recently, herbal preparations and natural compounds have been reported to possess antihepatocellular carcinoma effects [3, 4]. Liquiritigenin (LQ), separated from* Glycyrrhiza radix*, possesses various biochemical activations including anti-inflammatory, antihyperlipidemic, antiallergic, and estrogenic properties [5–8]. Its chemical structure and HPLC chromatograms in* Glycyrrhiza radix* water extraction are shown in Figure 1. As reported previously, LQ has inhibited cell proliferation in human lung fibroblasts, peripheral lymphocytes, SMMC-7721, and Hela cells [9, 10]. Fifteen-day LQ treatment suppresses murine H22-xenografted tumor growth in mice [11]. Our separate research indicates that LQ exhibits antitumor action in pituitary adenoma cells via Ras/ERKs and ROS-dependent mitochondrial signaling pathways [12]. However, there are no direct studies related to the cytotoxic effects of LQ on human hepatocellular carcinoma cells.

Apoptosis, a complex programmed cell death, is associated with various signaling [13]. Mitochondrial depolarization, associated with apoptotic cell death, is considered as a target for cancer therapy [14, 15]. B-cell lymphoma-extra large (Bcl-xL) and B-cell lymphoma 2 (Bcl-2), located in mitochondria, are believed to be central regulators for mitochondrial dysfunction [16]. Intracellular reactive oxygen species (ROS) level is another factor responsible for apoptosis and mitochondrial depolarization, which is a byproduct of cellular oxidative progresses [17]. Enhanced mitochondrial permeabilization causes excessive ROS release, further leading to mitochondria damage [18, 19]. On the other hand, a link between intracellular ROS accumulation and mitogen-activated protein kinase (MAPKs) activation is reported by ample previous studies [20, 21]. As reported previously, MAPKs pathway plays a significant regulatory role in cell proliferation, invasion, migration, and metastasis [22].

In the present study, we aim to investigate the* in vitro* and* in vivo* antitumor effects of LQ on hepatocellular carcinoma cells. Results revealed that LQ induced apoptotic cell death in HepG2 and PLC/PRL/5 cells mainly through MAPKs-mediated pathway. We further found that LQ-mediated mitochondrial dysfunction and MAPKs activation were associated with intracellular ROS accumulation. Our study indicates the potential of LQ on the list of possible agents for hepatocellular carcinoma treatment.

## 2. Material and Methods

### 2.1. Cell Culture

PLC/PRF/5 (p53 mutant) and HepG2 (p53 wild type) human HCC tumor cells were obtained from American Type Culture Collection (ATCC) and cultured in Dulbecco's Modified Eagle Media (DMEM) supplemented with 10% fetal bovine serum (FBS), 100 units/mL penicillin, and 100 *μ*g/mL streptomycin under a humidified atmosphere containing 5%/95% CO_2_/air at 37°C. The culture medium was changed every 3 days. Cell culture reagents were obtained from Invitrogen, USA.

### 2.2. Assessment of Cell Viability

Cells were plated into 96-well plates at 5,000 per well. On the following day, cells were incubated with 100 *μ*M–500 *μ*M LQ (Purchased from Source Leaf Biological Technology Co, LTD, Shanghai, China; Purity > 98.0%) for 24 h. LQ was dissolved in dimethyl sulfoxide (DMSO) and served as a stock solution. Cell viability was measured by a quantitative colorimetric assay with 3-(4,5-dimethylthiazol-2-yl)-2,5-diphenyltetrazolium bromide (MTT; Sigma-Aldrich, USA). The MTT assay quantifies mitochondrial dysfunction by measuring the formation of a formazan product. Briefly, treated cells were incubated with 0.5 mg/mL MTT for 4 h at 37°C in darkness. 100 *μ*L of DMSO was added to each well to solubilize purple formazan crystals. The absorbance was measured using a microplate reader at a wavelength of 540 nm (Bio-Rad, USA).

### 2.3. Assessment of Released Lactate Dehydrogenase (LDH)

LDH assay determines membrane integrity by measuring release of LDH in the culture medium.* In Vitro *Toxicology Assay Kit (Sigma-Aldrich, USA) was applied in our study. Cells were seeded into 96-well plates at 5,000 per well and incubated overnight. After exposure to 200 *μ*M and 400 *μ*M LQ for 24 h, cultured medium was collected. 60 *μ*L mixed assay solution was added to 30 *μ*L cultured medium. After incubation for 30 min at room temperature in darkness, 10 *μ*L 1 N HCl was added to terminate the reaction, followed with spectrophotometrically measured absorbance at a wavelength of 490 nm. LDH release was expressed as a percentage of corresponding unexposed cells.

### 2.4. Flow Cytometry Analysis of Cell Apoptosis

Cells were seeded into 6-well plates at 2 × 10^5^ per well. After exposure to 200 *μ*M and 400 *μ*M LQ for 24 h, cells were suspended in binding buffer (1 × 10^6^/mL) and incubated with 5 *μ*L annexin V-FITC (20 *μ*g/mL) and 5 *μ*L propidium iodide (PI; 50 *μ*g/mL) (Becton Dickinson Company, CA USA) for 10 min at room temperature in darkness. The stained cells were analyzed by flow cytometry directly (FC500, Beckman Coulter, USA) and the apoptosis rate was quantified using Flow Jo software. Living cells (annexin V−/PI−, Q4), early/primary apoptotic cells (annexin V+/PI−, Q3), late/secondary apoptotic cells (annexin V+/PI+, Q2), and necrotic cells (annexin V−/PI+, Q1) were identified and their percentages were calculated. The experiment was repeated three times.

### 2.5. Assessment of ROS

The intracellular ROS level was measured by 2′–7′-dichlorodihydrofluorescein diacetate (DCFH-DA, Sigma-Aldrich, USA) staining. Cells were seeded into 6-well plates at 2 × 10^5^ cells per well. After 12 h treatment with 200 *μ*M and 400 *μ*M of LQ, cells were suspended and incubated with 10 *μ*M DCFH-DA at 37°C for 10 min in darkness. After three washes with phosphate buffered saline (PBS), the changes of intracellular ROS level was analyzed by flow cytometry (FC500, Beckman Coulter, USA). The green fluorescence intensity was increased followed by intracellular ROS accumulation. The experiment was repeated for three times.

### 2.6. Assessment of Mitochondrial Membrane Potential (MMP, Δ*ψm*)

5,5′,6,6′-Tetrachloro-1,1′,3,3′ tetraethylbenzimidazolylcarbocyanine iodide (JC-1; Sigma-Aldrich, USA), which selectively enters mitochondria, is used to measure Δ*ψm* changes. PLC/PRL/5 and HepG2 cells were seeded into 6-well plates at 2 × 10^5^ cells per well. Treated cells were incubated with 2 *μ*M JC-1 at 37°C for 20 min in darkness. After three washes with PBS, the changes of fluorescent color in the mitochondria were analyzed by fluorescent microscopy (20x; CCD camera, Axio Observer Z1; Carl Zeiss, Germany). Red fluorescence indicates healthy cells with high MMP, whereas green fluorescence indicates apoptotic or unhealthy cells with low MMP. The average of fluorescence intensity was calculated with software Image J (*n* = 3) and data are expressed as the ratio of red to green fluorescent intensity.

### 2.7. Assessment of Activity of Caspase 3

Cells were planted into 6-well plates at 2 × 10^5^ cells per well. Treated cells were collected and lysed with RIPA buffer (Sigma, USA) with 1% protease inhibitor cocktail (Sigma-Aldrich, USA) and 2% phenylmethanesulfonyl fluoride (PMSF; Sigma-Aldrich, USA). Protein concentration was examined using Bio-Rad protein assays, and the activities of caspase 3 were detected by a caspase 3 colorimetric detection kit (Enzo Life Sciences International, Inc.) according to the manufacturer's protocol. The activities of caspase 3 were expressed as a percentage of corresponding unexposed cells in three separate experiments.

### 2.8. Western Blot

Cells were planted into 6-well plates at 2 × 10^5^ cells per well. The following day, cells were treated with doses of LQ at indicated times. For detection of migration of phosphor-ERKs from cytoplasm to nucleus, cytoplasmic and nucleic extracts were prepared according to the previous study by Yang et al. [23]. For whole cell lysates, cells were lysed by RIPA buffer (Sigma-Aldrich, USA) containing the 1% protease inhibitor cocktail (Sigma-Aldrich, USA) and 2% PMSF (Sigma-Aldrich, USA). 30 *μ*g proteins were separated using a 12% SDS-PAGE gel and transferred electrophoretically onto nitrocellulose membranes (0.45 *μ*m, Bio Basic, Inc.). The transferred membranes were then blotted with the following primary antibodies at 4°C overnight at dilution of 1 : 1000: phosphor-c-Jun N-terminal kinases (P-JNK), total-JNK (T-JNK), phosphor-P38 (P-P38), total-P38 (T-P38), phosphor-ERKs (P-ERKs), total-ERKs (T-ERKs), cleaved poly (ADP-ribose) polymerase (PARP), Bcl-2, and Bcl-xL, glyceraldehyde-3-phosphate dehydrogenase (GAPDH) (Cell Signaling Technology, Beverly, MA), followed by treatment with horseradish peroxidase-conjugated secondary antibodies (Santa Cruz, USA). Chemiluminescence was detected using ECL detection kits (GE Healthcare, UK). The intensity of the bands was quantified by scanning densitometry using software Quantity One-4.5.0.

### 2.9. PLC/PRF/5-Xenografted Tumor Model

5-week-old male BALB/c athymic nude mice were used for* in vivo* studies. Experimental protocol was approved by Jilin University. The mice were housed in groups of two in clear plastic cages and maintained on a 12 h light/dark cycle (lights on 07:00–19:00 h) at 23 ± 1°C with water and food available* ad libitum*.

Since it is hard to establish* in vivo* tumor model by HepG2 cells, PLC/PRL/5-xenografted tumor model was developed. Tumors were generated by harvesting PLC/PRF/5 cells from mid-log phase cultures. A volume of 0.1 mL (5 × 10^7^ cells/mL) of cell suspension was subcutaneously (s.c.) injected into the right side of the waist of each mouse. 3–5 days later, when tumor diameters reached 3–5 mm, the mice were divided into 2 groups (*n* = 3 each) randomly. Mice were administered 20 mg/kg of LQ (treated group) or 0.9% saline solution (vehicle group) intraperitoneally every other day continuously for 18 days. Treatment producing >20% net body weight loss was considered “toxic.” Body weights and tumor dimensions were measured every other day. Tumor volume (mm^3^) was estimated using the equation length × (width)^2^  × 0.5. The mice were sacrificed at the end of the experiment by administration of 200 mg/kg pentobarbital. Tumor tissues were carefully dissected from each mouse.

### 2.10. Statistical Analysis

All values were expressed as mean ± S.D. One-way variance analysis (ANOVA) was used to detect statistical significance followed by post hoc multiple comparisons (Dunn's test). *P* < 0.05 was considered statistically significant.

## 3. Results

### 3.1. Intracellular Toxic Effects of LQ in Hepatocellular Carcinoma Cells

**Figure 2:**
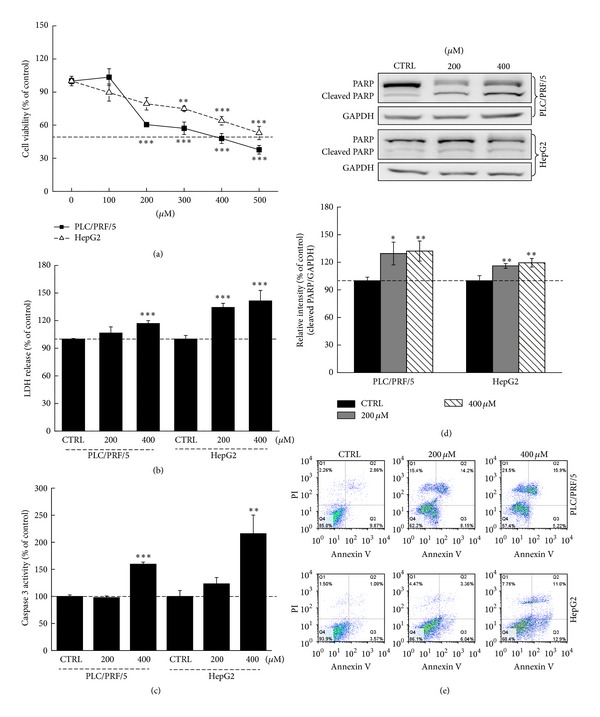
LQ induced cell damage in both HepG2 and PLC/RPL/5 cells. LQ dose-dependently reduced cell viability after 24 h treatment in hepatocellular carcinoma cells (a). 24 h LQ exposure resulted in an enhancement on LDH release (b) and caspase 3 activity (c). The expression of cleaved PARP was increased by presentation to LQ (200 *μ*M and 400 *μ*M) for 24 h and quantification data was normalized by GAPDH (d). LQ dose-dependently enhanced the apoptotic rate after 12 h incubation in the two cell lines (e). Data are expressed as percent of corresponding control cells and the means ± S.D. (*n* = 3). *P < 0.05, **P < 0.01, and ***P < 0.001 versus control cells.

LQ treatment results in a reduction in the cell viability in both HepG2 and PLC/PRF/5 cells and showed a dose- and time-dependent effect. The 24 h IC50 of LQ in PLC/PRF/5 and HepG2 were approximately 372.5 *μ*M and 495.5 *μ*M, respectively (Figure 2(a)). Compared with untreated cells, a 16.9 ± 3.1% and 41.4 ± 11.4% increase of intracellular LDH level was observed in PLC/RPL/5 and HepG2, respectively, after exposure to 400 *μ*M LQ for 24 h (Figure 2(b)). Increased LDH release has been observed in brain injury [24] and cells exposed to toxins [25]. The over-release of LDH may be responsible for LQ-mediated hepatocellular carcinoma cell damage. 24 h LQ (400 *μ*M) treatment enhanced the activities of caspase 3 in the two cell lines (Figure 2(c)). Moreover, a significant increment of the expression of cleaved PARP was observed after 24 h LQ (400 *μ*M) incubation (Figure 2(d)). Annexin V-FITC/PI double staining was performed to assess the rate of apoptotic cell death in both PLC/RPL/5 and HepG2 cells. Compared with unexposed cells, a significantly higher apoptotic rate in LQ-exposed cells was examined (Figure 2(e)). All these data confirmed that LQ showed cytotoxic effects in both HepG2 and PLC/PRF/5 cells.

### 3.2. LQ Caused Apoptotic Alteration on Mitochondrial Function and the Expressions of Bcl-2 and Bax

**Figure 3:**
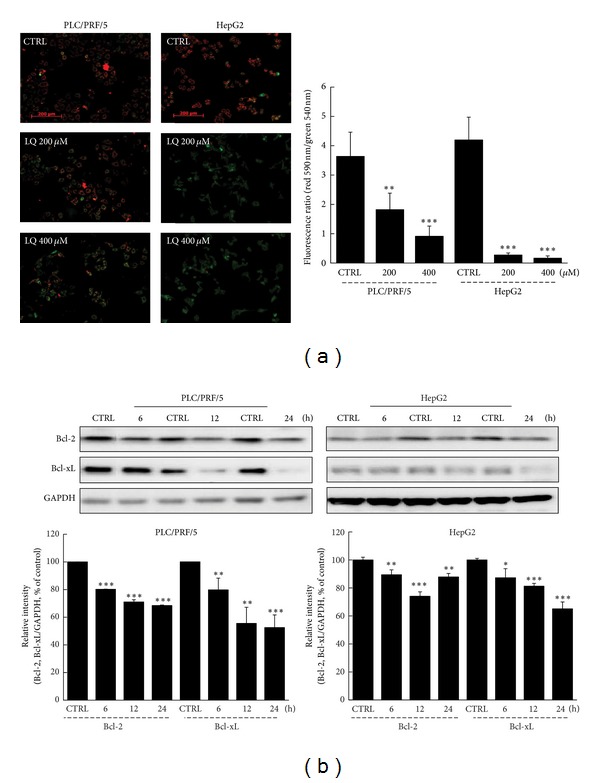
LQ caused the dissipation of Δ*ψm* and abnormal expression of Bcl-2 and Bcl-xL in hepatocellular carcinoma cells. (a) LQ (200–400 *μ*M) significantly caused the dissipation of Δ*ψm* which was detected by JC-1 staining. Qualification data were expressed as the ratio of red to green fluorescent intensity. (b) LQ (400 *μ*M) time-dependently reduced the expressions of Bcl-2 and Bcl-xL from 6 h to 24 h. Quantification data of the expressions of Bcl-2 and Bcl-xL were normalized by corresponding GAPDH and expressed as percent of corresponding control cells. Data are expressed as mean ± S.D. (*n* = 3) and analyzed using one-way ANOVA. *P < 0.05, **P < 0.01, and ***P < 0.001 versus control cells.

It has been reported that cell apoptosis is related to mitochondrial function [26]. JC-1 staining was performed to determine the changes of Δ*ψm*. LQ dose-dependently induced the dissipation of Δ*ψm* in both HepG2 and PLC/PRL/5 cells indicated by an increment in green fluorescence and reduction in red fluorescence (*P* < 0.01; Figure 3(a)).

Both Bcl-2 and Bcl-xL play important roles in mitochondrial function and cell survival [27]. LQ (400 *μ*M) strongly reduced the expressions of Bcl-2 and Bcl-xL in hepatocellular carcinoma cells from 6 h to 24 h (*P* < 0.05; Figure 3(b)). Collectively, LQ induced intracellular toxicity via mitochondrial function, which is associated with the expressions of Bcl-2 and Bcl-xL.

### 3.3. The Activation of MAPKs Was Involved in LQ-Mediated Cytotoxicity in Hepatocellular Carcinoma Cells

**Figure 4:**
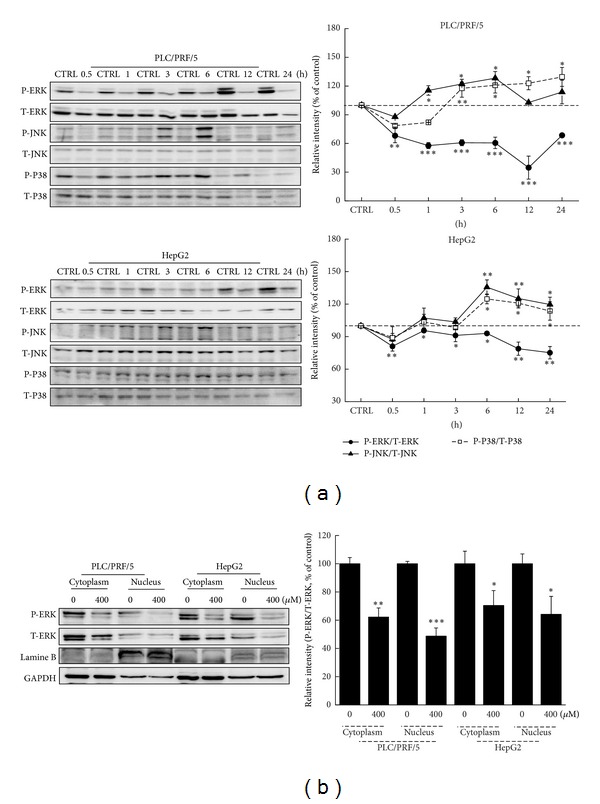
The activation of MAPKs contributes to LQ-mediated apoptotic cell death. (a) 400 *μ*M LQ treatment resulted in a reduction of the expression of P-ERKs and an increase of the activation of JNK and P38 from 0.5 h to 24 h treatment. (b) The migration of P-ERKs from cytoplasm to nucleus was suppressed by LQ after 3 h exposure. Quantification data of the expressions of P-ERKs, P-JNK, and P-P38 were normalized by corresponding T-ERKs, T-JNK, and T-P38. Data are expressed as mean ± SD (*n* = 3) and analyzed using one-way ANOVA. *P < 0.05, **P < 0.01, and ***P < 0.001 versus untreated cells.

MAPKs are believed to play key roles in mechanisms in cell proliferation, differentiation, survival, and apoptosis [20]. Results revealed that LQ time-dependently suppressed the phosphorylation of ERKs (*P* < 0.05; Figure 4(a)) from 0.5 h to 24 h; meanwhile, after 3 h LQ (400 *μ*M) treatment, P-ERKs nucleus translocation was suppressed (*P* < 0.05; Figure 4(b)). Furthermore, 400 *μ*M LQ treatment strongly enhanced the activations of JNK and P38 in hepatocellular carcinoma cells (*P* < 0.05; Figure 4(a)). These data suggest that the activations of MAPKs contribute to the LQ-mediated antihepatocellular carcinoma effect.

### 3.4. Intracellular ROS Accumulation Played a Crucial Role in LQ-Mediated Apoptotic Cell Death

**Figure 5:**
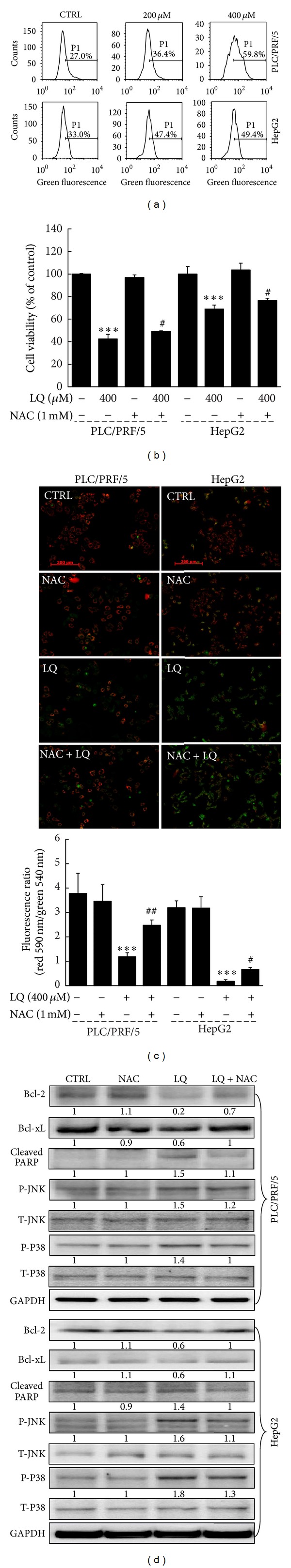
Intracellular ROS accumulation displayed an important role in LQ-mediated cytotoxicity in hepatocellular carcinoma cells. (a) LQ dose-dependently enhanced intracellular ROS level after 12 h treatment. (b) NAC pretreatment strikingly abrogated LQ-reduced cell viability. (c) LQ-induced the dissipation of Δ*ψm* was partially restored by NAC pretreatment. Qualification data were expressed as the ratio of red to green fluorescent intensity (*n* = 3). (d) LQ-induced apoptotic alteration on the expression of antiapoptosis and proapoptosis related proteins was strikingly reversed by NAC pretreatment. The average fold of band intensity compared with CTRL group was marked, respectively (*n* = 3). Cells were pretreated with 1 mM NAC for 30 min and then cotreated with 400 *μ*M LQ for another 24 h (b) or 12 h ((c) and (d)). Data are expressed as mean ± S.D. (*n* = 3) and analyzed using one-way ANOVA. ***P < 0.001 versus control cells, ^#^ *P* < 0.05, and ^##^ *P* < 0.01 versus LQ-treated cells.

Intracellular ROS was emphasized in ample previous studies for its important role in inducing cell death [28]. After exposure to 200 *μ*M and 400 *μ*M LQ for 12 h, a robust increment of intracellular ROS level was observed, exhibiting the potential role of ROS production inducing hepatocellular carcinoma cell death (Figure 5(a)). Additionally, after pretreatment with 1 mM* N*-acetyl-L-cysteine (NAC) for 30 min and cotreatment with 400 *μ*M LQ for 24 h, LQ-reduced cell viabilities were alleviated by NAC significantly (*P* < 0.05; Figure 5(b)).

It is believed that hyperlevel of intracellular ROS is responsible for mitochondrial dysfunction [29]. Our results revealed that 1 mM NAC pretreatment strongly restored Δ*ψm* loss caused by LQ, indicated by the enhancement of red fluorescence (*P* < 0.05; Figure 5(c)).

Additionally, ROS production causes sustained MAPKs activation [21]. LQ-enhanced activations of JNK and P38 were abrogated in cells exposed to 1 mM NAC for 30 min and then incubated with 400 *μ*M LQ for 12 h (Figure 5(d)). Moreover, data revealed that NAC strikingly restored LQ-induced apoptotic alteration on the expressions of Bcl-2, Bcl-xL and cleaved PARP (Figure 5(d)). Collectively, our data indicated that intracellular ROS accumulation was involved in LQ's antitumor effect.

### 3.5. LQ Inhibited PLC/RPL/5-Xenografted Tumor Growth in Nude Mice

**Figure 6:**
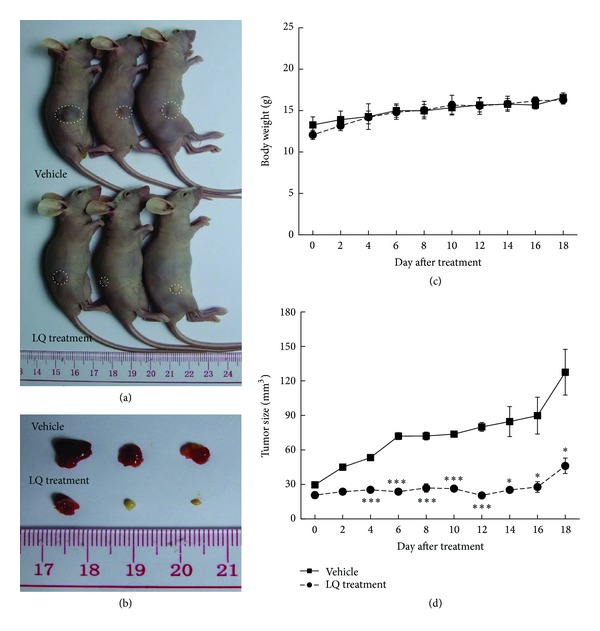
LQ inhibited PLC/PRF/5-xenografted tumor growth in BALB/c nude mice. Six BALB/c athymic nude mice inoculated with PLC/PRL/5 cells (5 × 10^6^ cells/mouse, i.p.) were treated with LQ (20 mg/kg, i.p., every other day) or vehicle solvent (0.9% saline solution, i.p., every other day) for 18 days. ((a), (b)) Tumor-possessing nude mice and tumor tissues separated from vehicle and LQ-treated groups. (c) Mean (S.D.) body weight of GA-treated and vehicle group (*n* = 3). (d) Tumor volumes were measured every other day. Tumor size in the curve was expressed as mean ± S.D. (*n* = 3). *P < 0.05 and ***P < 0.001 versus vehicle cells.

In the PLC/RPL/5-xenografted tumor nude mice model, tumor growth inhibition was most evident in mice treated with 20 mg/kg LQ (every other day), where nearly 65% reduction in tumor size was observed compared with vehicle mice (*P* < 0.05; Figures 6(a), 6(b), and 6(d)). Meanwhile, LQ administration showed little effect on body weight (Figure 6(c)). The* in vivo* data further documented the LQ-mediated antihepatocellular carcinoma effect.

## 4. Discussion

Data from our group and others have already demonstrated the cytotoxic effects of LQ in pituitary adenoma cells, Hela cells [30], SMMC-7721 cells [10], human lung fibroblasts, and peripheral lymphocytes [9]. In our present study, the potential antitumor effect on hepatocellular carcinoma was investigated in* in vitro* and* in vivo* models. LQ possesses robust cytotoxic effects in both PLC/PRL/5 and HepG2 cells, as evidenced by decreased cell viability, increased LDH release and increased caspase 3 activity, causing cellular and mitochondrial apoptotic alterations. Our series of experimental data reveal that the activations of MAPKs contribute to LQ-mediated antihepatocellular carcinoma effects; moreover, LQ-enhanced intracellular ROS level is responsible for mitochondrial dysfunction and MAPKs activation.

Ample studies suggest that mitochondrial function is responsible for cell death and is considered a target for anticancer drugs [31–33]. We confirm that LQ enhances the dissipation of Δ*ψm* in hepatocellular carcinoma cells. Meanwhile, the reduction in Bcl-2 and Bcl-xL expression was noted. Bcl-2 family members are essential mediators which are located in the outer mitochondrial membrane [34]. Reduction in their expression causes mitochondrial apoptotic alteration and further results in MMP loss [35]. Thus, the cytotoxic effects of LQ are at least partially attributed to its modulation of mediators associated with mitochondrial function.

On one hand, free radical accumulation is an essential factor contributing to cell damage and mutation; meanwhile, intracellular ROS accumulation is responsible for mitochondrial membrane permeability which is reported by ample studies [29, 36]. LQ treatment results in an increase in ROS concentration; consequently, NAC partially ameliorates LQ-induced Δ*ψm* loss and apoptotic alteration on the expressions of Bcl-2 and Bcl-xL. Altogether, hyper-level of intracellular ROS contributes to LQ-modulated mitochondrial dysfunction. Furthermore, ROS production causes sustained MAPKs activation which has been studied intensively as a possible mechanism for cell proliferation, differentiation, survival, and apoptosis [20, 21]. NAC not only reverses LQ-reduced cell viability but also suppresses JNK and P38 activation enhanced by LQ. As reported previously, mitochondrial function is associated with the phosphorylation of JNK and P38 [37, 38]. Our present data demonstrated that ROS may serve as a link between mitochondrial dysfunction and JNK and P38 activation; however, further experiments must be performed to confirm this hypothesis.

The ERKs pathway is upregulated in human tumors and, as such, represents an attractive target for anticancer therapy [39, 40]. The phosphorylation of ERKs results in their nuclear translocation and activation of numerous substrates, promoting proliferation and inhibiting proapoptotic signals [41]. In our experiment, LQ significantly reduced the phosphorylation of ERKs in both PLC/PRL/5 and HepG2 cells and further reduced the migration of phospho-ERKs from cytoplasm to nucleus. One study has indicated that modulating the localization of ERKs in the nucleus is responsible for colorectal cancer cell apoptosis [42]. Additionally, ERKs inhibitor downregulated the expression of Bcl-2 and Bcl-xL [43], and ERKs/Bcl-2 signaling is believed to be a potential therapeutic target for cancer cells [44, 45]. Collectively, the activation of ERKs contributes to LQ-caused Δ*ψm* loss. LQ-mediated hepatocellular carcinoma cell death is closely associated with the ERKs signaling pathway.

**Figure 7:**
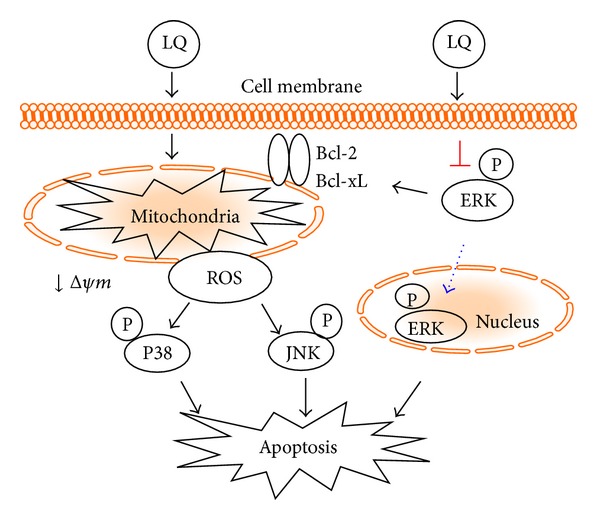
Schematic illustration of putative mechanisms of LQ-induced apoptotic cell death.

In summary, our present study confirms the antihepatocellular carcinoma effect of LQ both in* in vitro* and* in vivo* experiments. LQ causes ROS increment, LDH over-release, Δ*ψm* dissipation, and abnormal expression of apoptosis related proteins. The activation changes of MAPKs contribute to LQ-induced cytotoxicity in hepatocellular carcinoma cells. Moreover, incremental ROS level serves as a link between the dissipation of Δ*ψm* and the phosphorylation of JNK and P38 (Figure 7). These findings provide pharmacological evidence that LQ possesses antihepatocellular carcinoma effects as a potential chemotherapeutic agent.

## Supplementary Material

The intracellular Ca^2+^ concentration which is responsible for cell apoptotic death also detected in our experiment. Results from Fluo4 AM staining revealed that exposure to LQ for 12 h dose-dependently increased the intracellular Ca^2+^ concentration. The morphology changes can also be detected. Shrinkage and detachment were observed after LQ treatment compared with control.
