# Supplementary material for: Liquiritigenin Induces Tumor Cell Death through Mitogen-Activated Protein Kinase- (MPAKs-) Mediated Pathway in Hepatocellular Carcinoma Cells
Source: Biomed Res Int. 2014 Mar 11;2014:965316. doi: 10.1155/2014/965316 (PMC3967596; doi:10.1155/2014/965316)
Supplement: Supplementary file 1 — The intracellular Ca2+ concentration which is responsible for cell apoptotic death also detected in our experiment. Results from Fluo4 AM staining revealed that exposure to LQ for 12 h dose-dependently increased the intracellular Ca2+ concentration. The morphology changes can also be detected. Shrinkage and detachment were observed after LQ treatment compared with control. [file 965316.f1.doc]

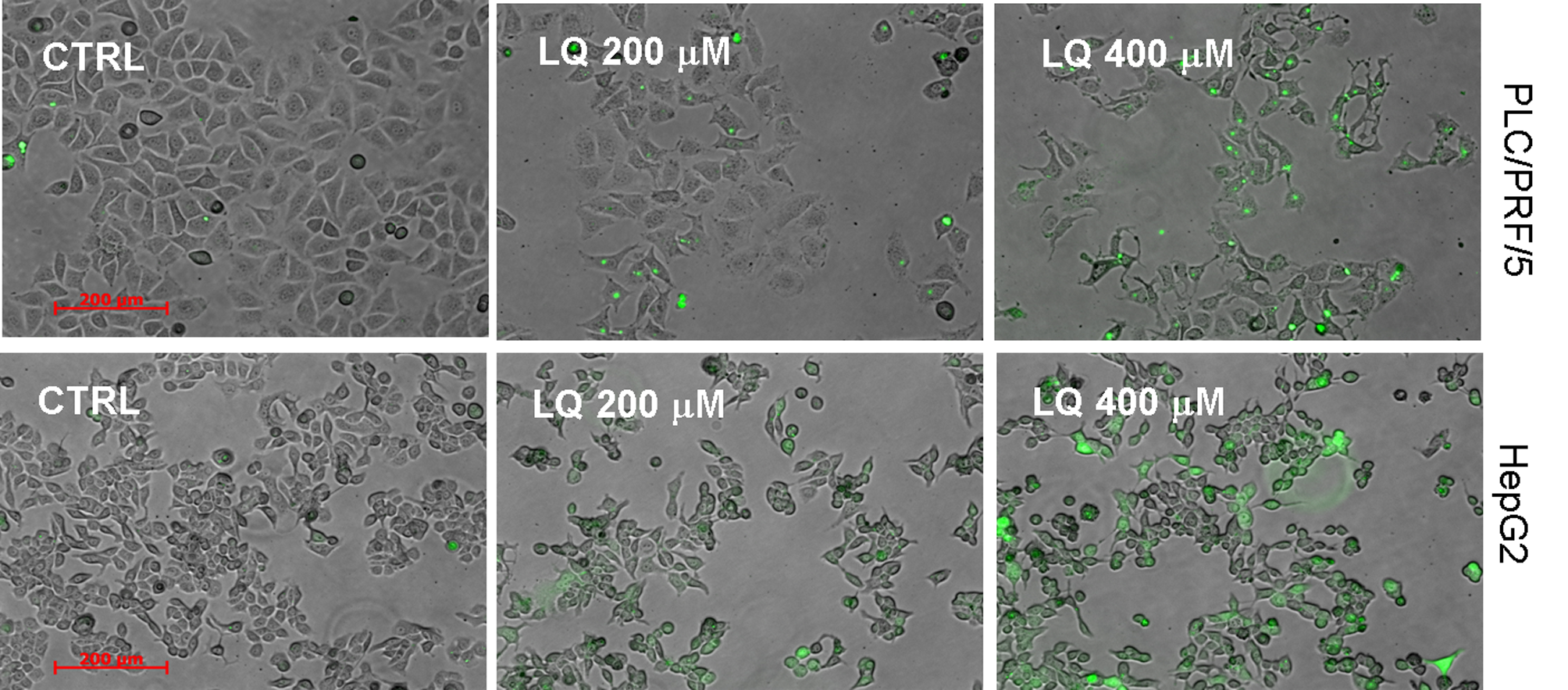


Fig.1s. LQ treatment enhanced the intracellular Ca^2+^ level and caused apoptotic changes in cell morphology.
